# Culture of Airway Epithelial Cells from Neonates Sampled within 48-Hours of Birth

**DOI:** 10.1371/journal.pone.0078321

**Published:** 2013-11-04

**Authors:** David Miller, Steve W. Turner, Daniella Spiteri-Cornish, Neil McInnes, Alison Scaife, Peter J. Danielian, Graham Devereux, Garry M. Walsh

**Affiliations:** 1 Department of Child Health, Royal Aberdeen Children’s Hospital, University of Aberdeen, Aberdeen, United Kingdom; 2 Institute of Medical Science, University of Aberdeen, Aberdeen, United Kingdom; 3 Aberdeen Maternity Hospital, Aberdeen, United Kingdom; National Jewish Health, United States of America

## Abstract

**Introduction:**

Little is known about how neonatal airway epithelial cell phenotype impacts on respiratory disease in later life. This study aimed to establish a methodology to culture and characterise neonatal nasal epithelial cells sampled from healthy, non-sedated infants within 48 hours of delivery.

**Methods:**

Nasal epithelial cells were sampled by brushing both nostrils with an interdental brush, grown to confluence and sub-cultured. Cultured cells were characterised morphologically by light and electron microscopy and by immunocytochemistry. As an exemplar pro-inflammatory chemokine, IL-8 concentrations were measured in supernatants from unstimulated monolayers and after exposure to IL-1β/TNF-α or house dust mite extract.

**Results:**

Primary cultures were successfully established in 135 (91%) of 149 neonatal samples seeded, with 79% (n  =  117) successfully cultured to passage 3. The epithelial lineage of the cells was confirmed by morphological analysis and immunostaining. Constitutive IL-8 secretion was observed and was upregulated by IL-1β/TNF-α or house dust mite extract in a dose dependent manner.

**Conclusion:**

We describe a safe, minimally invasive method of culturing nasal epithelial cells from neonates suitable for functional cell analysis offering an opportunity to study “naïve” cells that may prove useful in elucidating the role of the epithelium in the early origins of asthma and/or allergic rhinitis.

## Introduction

The airway epithelium is structurally and functionally abnormal in asthma and is thought to play critical roles in the initiation, orchestration and perpetuation of immune and inflammatory responses resulting in the airway inflammation and remodeling characteristic of asthma [1]. Compared to healthy subjects there is evidence that the asthmatic epithelium has an increased susceptibility to injury and an impaired ability to repair itself [2], [3], with exaggerated release of pro-inflammatory mediators, both constitutively [3]–[7] and in response to environmental stress [8].

In recent years it has become increasingly evident that antenatal factors modulate the likelihood of childhood asthma [9], [10]. For example, reduced fetal size at 10 weeks gestation is associated with an increased likelihood of asthma, wheezing symptoms and impaired lung function in 5 year old children [11], whilst reduced lung function within days of birth is associated with reduced lung function and increased wheezing and asthma by 10–11 years of age [12]. It has been postulated that antenatal exposures, including maternal intake of certain nutrients [13] and cigarette smoking during pregnancy [14], predispose to airway disease by impacting on fetal airway development. Such epidemiological considerations suggest that airway epithelial cell (AEC) function may be abnormal from the earliest stages of airway organogenesis and that AEC function is intrinsically different at the time of birth in children who develop asthma in later life [1]. Moreover, it is plausible that antenatal exposures might impact on AEC development and be associated with neonatal AEC function.

In adults and children, AEC from the lower respiratory tract can be obtained by bronchoscopic or ‘blind’ brushings of the airway mucosa. However practical and ethical issues prohibit such an approach for research purposes in new born infants. In contrast, the nose is a relatively non-invasive and readily accessible source of AEC. We previously investigated the potential to use nasal AEC as surrogates for bronchial AEC in adults and children [15], [16] and have therefore developed a method to harvest and culture airway epithelial cells from neonates within the first two days of life. Such an approach permits the study of essentially “naïve” airway cells not yet exposed to the modifying effects of inhaled environmental pollutants and pathogens. Neonate nasal AEC potentially offer a unique tool to gain insight into the role of antenatal influences on airway development and the pathogenesis of asthma and allergic rhinitis. We describe here our initial experiences of successfully sampling and culturing neonatal nasal epithelial cells.

## Materials and Methods

### Subjects and study design

Mothers were recruited before delivery to either a small pilot project or as part of a larger birth cohort study. For the pilot work, women were enrolled from a dedicated elective Caesarean section clinic the week before delivery. For the cohort study, healthy unselected pregnant women clinically triaged to low risk antenatal care attending a routine ‘dating’ ultrasound scan clinic appointment at Aberdeen Maternity Hospital at 10–12 weeks gestation were invited to participate in the cohort study. Women were recruited regardless of their smoking status or personal or family history of asthma or atopic disease.

### Ethics statement

Written informed consent was obtained from parents and basic demographic data recorded. The study was approved by the North of Scotland Research Ethics Service, Scotland, UK (10/S0802/55).

### Sampling of nasal epithelial cells

**Figure 1:**
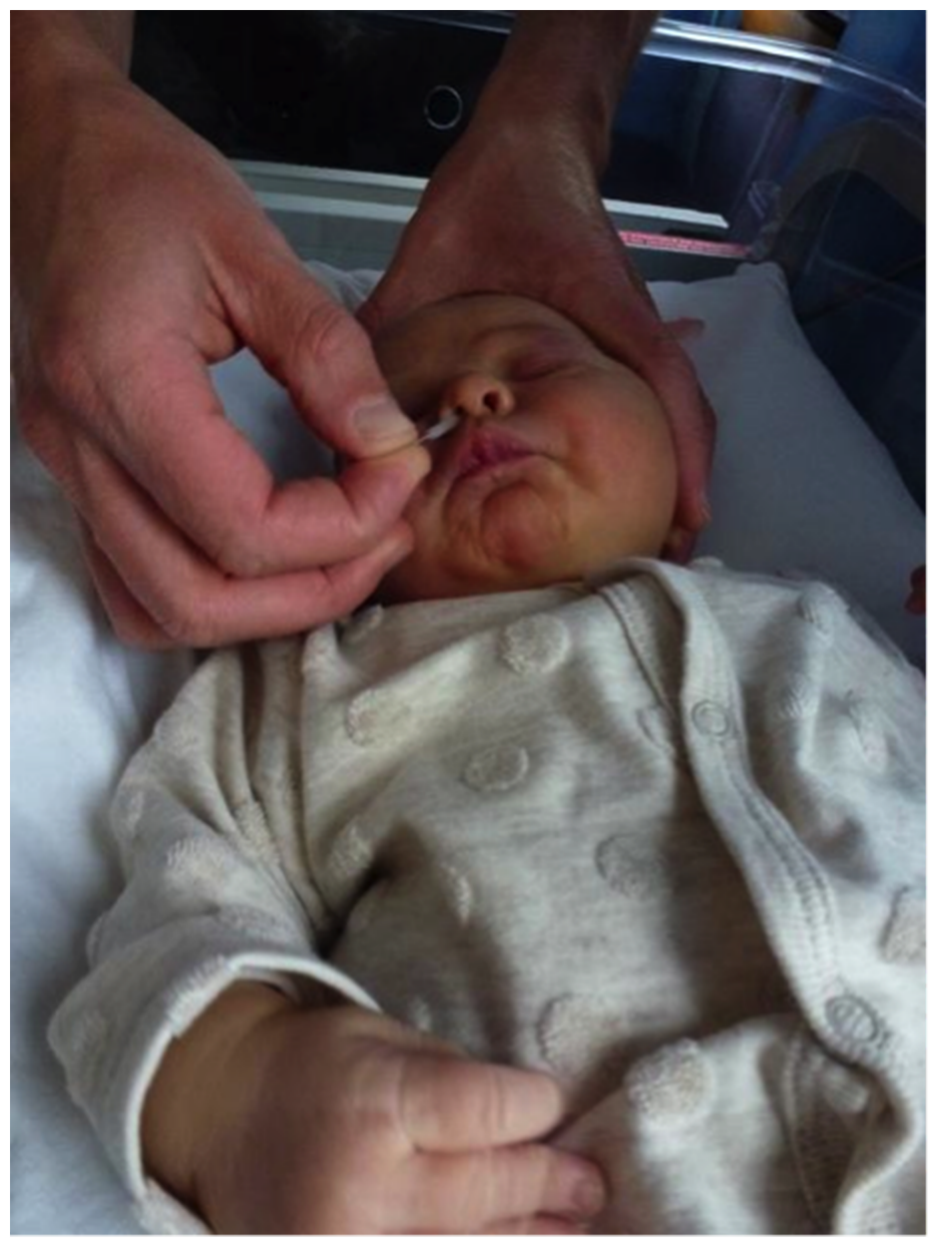
Technique for harvesting nasal epithelial cells from newborn infants. Written informed consent, as outlined in the PLOS consent form, to publication of this photograph was given by the child’s legal guardian.

Nasal AEC were harvested from healthy, non-sedated neonates born to the recruited women at the earliest opportunity after birth. Samples were taken if the baby was born after 36 weeks gestation, there were no health concerns (e.g. a significant perinatal complication, maternal group B streptococcus colonisation) and the mother provided further verbal consent. Sampling was undertaken either with the infant lying in their parents arms or in a cot. After gently securing the infants head with one hand a 2.7mm diameter interdental brush (DentoCare Professional, London, UK) was gently introduced into each nostril in turn to obtain cells from the medial aspect of the inferior turbinate. Using the same brush, a single pass of both nostrils was performed (Figure 1).

### Sample Processing

Nasal AEC were cultured, stimulated, phenotyped and mediator release quantified as previously described [15], [16]. Briefly, immediately after collection the sampling brush was agitated to detach cells from the brush tip into an Eppendorf containing 1ml of specific serum-free bronchial epithelial growth media (Lonza Ltd., Slough, UK) supplemented with 1% penicillin/streptomycin. The detached cells were then used to establish nasal AEC cultures in all but 6 samples which were used for either cell count or cytospin preparations (n = 3 for both). Cytospins were subsequently evaluated microscopically by a senior consultant cytopathologist.

For culture**,** each sample was seeded into a sterile plastic 6-well plate pre-coated with collagen with a growth area of 9.6 cm^2^ (Greiner Bio-One Ltd, Stonehouse, Gloucestershire, UK) containing a further 2ml of growth media within 30 minutes of harvesting. Monolayers were passaged with trypsin at 70–90% confluence. Cells were incubated at 37°C in 5% CO_2_/95% air with media changed at 24 hours after seeding or passaging and thereafter replaced with fresh media every 3 days. Confluent tertiary passage cells seeded at a density of 10^4^ cells/cm^2^ were used in all experiments unless otherwise stated.

### Immunocytochemistry

Cells were grown on chamberslides, fixed in 4% formaldehyde, washed then permeabilsed with 0.1 Triton X-100 before blocking non-specific binding sites with 10% fetal calf serum and 0.3 M glycine in PBS. Primary antibodies against cytokertain 19 and 13 (both Vector Laboratories, Peterborough, UK), as well as an istotype matched control antibody at equivalent concentrations were then applied overnight at 4°C. Cells were then incubated with a secondary antibody coupled to Alexa488 for 1 hour at room temperature before mounting with mounting media containing DAPI (Vector Laboratories, Peterborough, UK). Imaging was performed with a Zeiss LSM 710 confocal microscope.

### Electron Microscopy

For transmission electron microscopy, cultured cells were fixed in 2.5% glutaraldehyde in 0.1 M phosphate buffer, post-fixed in 1% osmium tetroxide, and then processed and embedded in TAAB 812 epoxy resin then embedded. Ultrathin sections (70 nm) were cut and then stained with uranyl acetate and lead citrate for examination at 80 kV using a Philips CM10 transmission electron microscope. Samples were prepared in an identical fashion for scanning electron microscopy, and after ethanol dehydration dried using hexamethyldisilazane (HMDS). Thereafter, dried samples were coated in gold using a EMitech K550 sputter coater and viewed in a Zeiss EVO MA10 scanning electron microscope at 10 kV.

### Constitutive and stimulated IL-8 release

Confluent nasal AEC monolayers in 3.5 cm^2^ wells were stimulated with house dust mite extract (HDM) (Greer Laboratories, Lenoir, NC, USA) or, as in our previous studies, interleukin 1β (IL-1β) in combination with tumor necrosis factor-α (TNF-α) (R&D, Abingdon, UK) for 24 hr, or left unstimulated [15], [16]. As before, IL-1-β and TNF-α were selected as both are implicated in the pathogenesis of many acute and chronic infectious and non-infectious inflammatory diseases of the lung, often acting synergistically.

Initially dose titrations studies were conducted in samples from 10–15 neonates. AEC monolayers were stimulated with IL-1β and TNF-α both at concentrations of 1, 10, 100 ng/ml or with house dust mite extract (HDM) at concentrations of 5, 10, 25 and 50 µg/ml. Monolayers were exposed to either media supplemented with stimulant or media alone for 24 hours before removal of the supernatant layer. AEC release of interleukin- 8 (IL-8) was used as an exemplar cytokine. Concentrations of IL-8 were measured in culture supernatants by ELISA as per the manufacturer’s instructions (Biolegend, San Diego, California, USA). Results were normalized to cellular protein content of lysed monolayers quantified using the Bradford assay as previously described [15].

### Statistical analysis

All statistical analyses were performed using IBM SPSS Statistics for Windows, Version 20.0. (Armonk, NY). IL-8 was detectable in the supernatants of all viable cultures, and the distribution of IL-8 approximated to a log-normal distribution. Parametric analyses (paired t tests, repeated measures ANOVA) were conducted on log_e_ transformed IL-8 values, with geometric means and 95% confidence intervals being computed.

## Results

### Sampling and culture establishment

Over a 28-month period, nasal AEC were harvested from 155 neonates (73 males) within the first two days of life (median time after birth to obtaining sample: 22.5 hours, IQR 14.8–31.3). 86 (55.5%) of those sampled were delivered by spontaneous vaginal delivery, the remainder born by Caesarean section (23.8%), Forceps (16.8%) or Ventouse (3.9%) delivery. All but 6 of the 155 neonates sampled were recruited to the cohort study. Antenatal and perinatal information on these pregnancies was collected and is summarized in Table 1.

**Table 1:** Antenatal and delivery data of sampled neonates (n = 149) recruited to the cohort study.

Mean maternal age at recruitment (range)	30.8 years (18–40)
Number of previous live births	
0	84 (56.4%)
1	47 (31.5%)
2 or more	18 (12.1%)
Never smoker	86 (57.7%)
Ex-smoker	55 (36.9%)
Current smoker	8 (5.4%)
Asthma	39 (26.2%)
Eczema	25 (16.8%)
Hayfever	54 (36.2%)
Mean gestation (range)	282 days (265-296)
Mean birth weight (range)	3590 grams (2430- 690)
Mean Occipital - frontal circumference (range)	34.8 cms (31- 8)

The nasal brushing took about 2 seconds for each nostril and was performed unaided by one of the research team (DM). Some of the infants cried, but stopped almost immediately when nursed by a parent. There were no adverse events and brushing was not associated with overt bleeding. Light microscopy examination of cytospins of three nasal samples by a senior cytopathologist revealed minimal erythrocytes. The number of cells harvested from the 3 samples pooled from each nostril used to quantify cell number ranged from 13×10^3^ to 55×10^3^ cells.

Initial samples were used to develop the culture methodology, to characterise the cultured cells and to establish optimal doses to stimulate the cultured cells for a subsequent observational epidemiological study. Of 149 samples seeded, primary monolayers were established from 91% (n = 135) neonates, with 79% (n = 117) of the total successfully cultured to confluence at passage 3. Nine of the unsuccessful cultures succumbed to infection. In the remaining cases, no obvious reason for failure of cells to grow could be identified (Table 2).

**Table 2:** Characteristics of cell growth by passage.

	Passage 1	Passage 2	Passage 3
**Success rate (% of 149 samples)**	135 (91%)	133 (89%)	117 (79%)
**Median time (IQR) to confluence**	11 days (8–14)	11 days (8–14.5)	14 days (8–20)

### Nasal AEC morphology and immunocytochemistry

**Figure 2:**
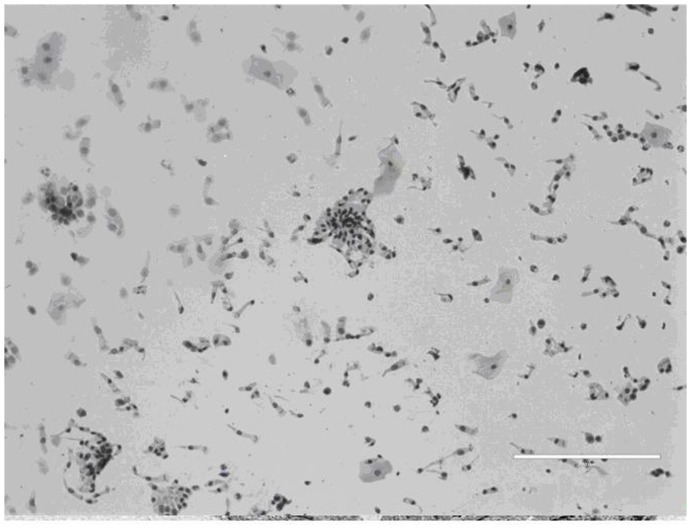
Cytospin of nasal sample obtained by brushing (size marker  =  200 µm).

**Figure 3:**
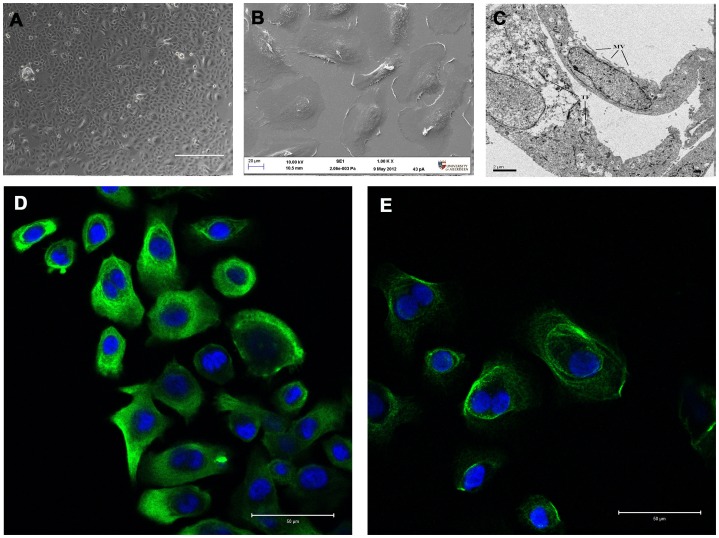
Appearance of cultured nasal epithelial cells at passage 3. (A) Phase contrast light micrographs showing typical “cobblestone” morphology of epithelial cells (size marker  = 400 µm). (B) Scanning and (C) transmision electron micrographs (size marker  =  20 µm and 2 µm respectively). Microvilli (MV) and tonofilaments (TF) indicated within cell ultrastucture. (D) and (E) positive immunostaining for cytokertain 19 and cytokeratin 13 respectively (green) and DAPI (blue) (size marker  =  50 µm).

Light microscopy of cytospins revealed a high (>95%) yield of airway epithelial cells consisting of ciliated epithelial cells and a subpopulation of non-ciliated cuboidal cells consistent with basal epithelial cells. Occasional squamous cells and goblet cells were also noted (Figure 2). Phase contrast microscopic evaluation of cultured cells demonstrated morphologic appearances consistent with epithelial lineage. By electron microscopy, cells had a flattened appearance with clearly identifiable nuclei and microvilli on the surface. The cytoplasm contained the usual range of organelles and included tonofilaments, characteristic of epithelial cells. Junctional complexes were not identified although some structures reminiscent of hemi-desmosomes were noted. AEC showed positive immunostaining for the epithelium-specific protein cytokeratin 19 and the basal epithelium marker cytokeratin 13 (Figure 3). Immunostaining with an isotype control primary antibody demonstrated no immunoreactivity.

### Response of cultured nasal AEC to stimulation with pro inflammatory cytokine and allergen

**Figure 4:**
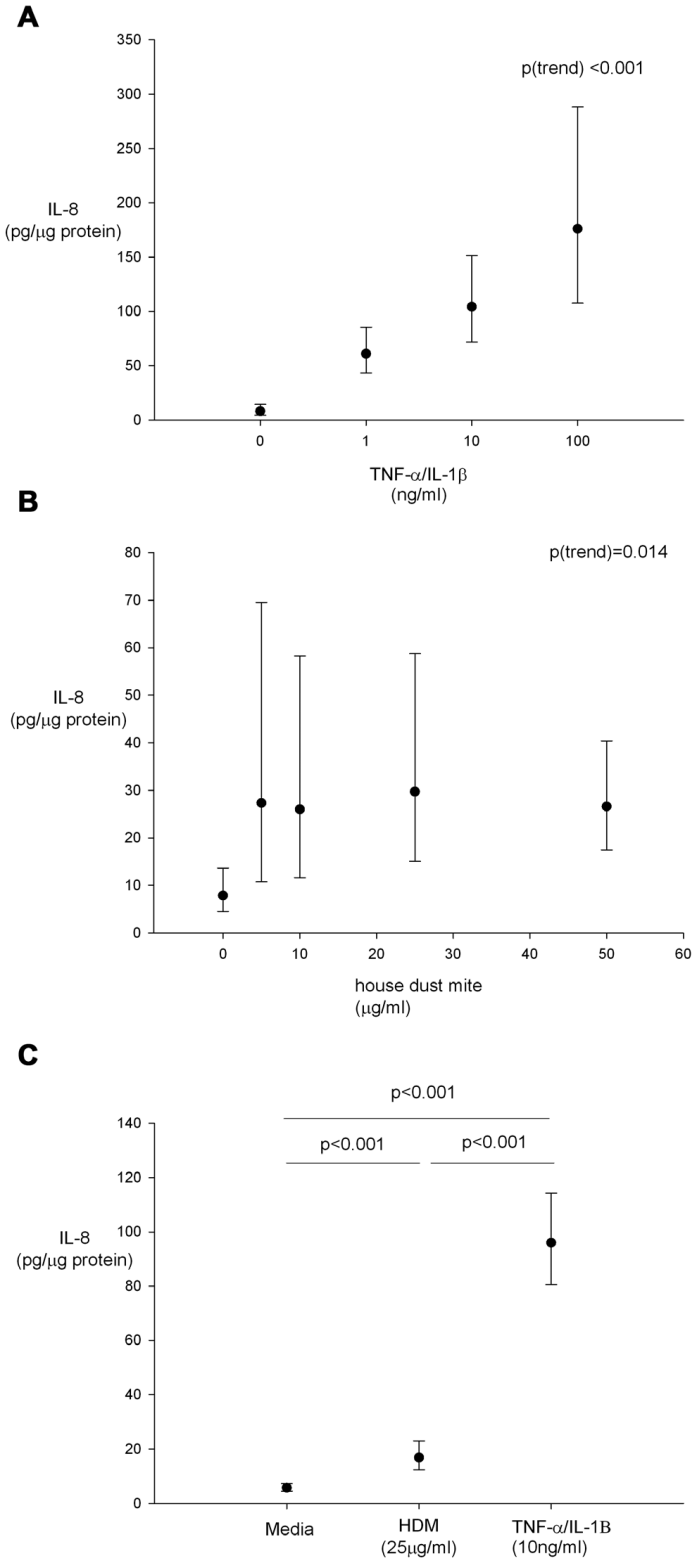
Geometric mean (95% CI) IL-8 responses of nasal AEC incubated with (A) increasing concentrations of TNF-α/IL-1β (n = 15), (B) increasing concentrations of HDM (n = 10) and (C) media, HDM (25 µg/ml) and IL-1β and TNF-α (both at 10 ng/ml) for 24 hours (n = 52). Repeated measures ANOVA and paired t-tests for statistical significance.

Dose titration experiments of cultured nasal AEC from 15 neonates stimulated with increasing concentrations of IL-1β and TNF-α demonstrated a dose dependent increase in IL-8 release (Figure 4a). Dose titration experiments were then conducted stimulating cultures from 10 neonates with increasing concentrations of house dust mite that induced a dose dependent increase in IL-8 release (Figure 4b), whether this was linear or quadratic was not clear. Subsequently AEC cultures from 52 neonates were stimulated with IL-1β & TNF-α (both at 10 ng/ml) and HDM at 25 µg/ml for 24 hours with IL-8 responses to TNF-α/IL-1β exposure being greater than for HDM at these concentrations (Figure 4c).

## Discussion

We are not aware of any published studies to date investigating neonate nasal AEC phenotype sampled and cultured within hours of delivery when the first and possibly most critical encounters occur between the newborn airway and the post natal environment. In this study we have shown that the sampling of nasal AEC from unsedated newborn infants is safe and well tolerated by the infant and acceptable to mothers. Cultured cells had the microscopic appearances and immunofluorescent features of AEC. Exposure of cultured neonate nasal AEC to TNF-α/IL-1β or HDM resulted in the release of IL-8 in a dose dependent manner. Over a relatively brief period of time, a significant number of samples were collected. Each nasal brushing took only a few seconds to perform and was not associated with any adverse effects. Our culture success rate suggests that the technique is reliable and could be used in large cohort studies.

There are reports of sampling nasal epithelial cells from infants in the literature; however the present study differs from these in several important respects. The case report of Lee et al [17] described serial nasal brushings from an infant from the age of 4 months, but the cells were not cultured. Similarly, O'Callaghan et al brushed the noses of 30 newborn children within 5 days of delivery for studies of ciliary beat frequency, again cells were not cultured [18]. One study did examine nasal AEC cultured from 15 infants [19]. However, the youngest infant was a month old, they were ‘held tightly by a nurse’ and because they were sedated with paracetamol and rectal midazolam they had to be monitored for 8 hours. This study also reported a single episode of epistaxis in their 15 subjects. In contrast, we observed no epistaxis in 155 neonates, this being most likely due to the smaller diameter of our sampling brush (2.7 mm), compared with the 5.5 mm brush used in the previous study. The paucity of erythrocytes identified in the cytospin preparations would suggest that the sampling procedure is relatively atraumatic in our hands.

Our methodology for sampling nasal AEC by nasal brushing in non-sedated neonates could prove a useful research tool and is especially applicable to large longitudinal studies. In addition to investigating the effect of *in-utero* influences on AEC phenotype, procuring neonatal airway epithelial tissue for culture has a variety of potential applications. As well as functional studies in submerged monolayers, it is likely that that our method could be adapted to grow neonatal nasal AEC in air-liquid interface cultures, which are thought to be more representative of conditions *in vivo,* this work is underway. Furthermore, obtaining nasal airway epithelial cells shortly after birth represents a privileged source of “naïve” tissue not yet exposed to the modifying effects of post natal environmental pollutants and pathogens. As such, the tissue specific epigenetic effects of such exposures throughout life on the expression of genes involved in inflammatory mediator release could be explored. Based on our previous success culturing nasal airway epithelial cells from both adults and children [15], [16], a study investigating repeated isolation of nasal cells from the same individual over time would be feasible. If neonatal nasal AEC are shown to be associated with subsequent asthma/allergic rhinitis and recognized/putative risk factors for these conditions, it may be possible to use neonatal nasal AEC as a neonatal biomarker in antenatal intervention studies. In the clinical setting it may be possible to use neonatal nasal AEC as a predictive biomarker to quantify subsequent risk of asthma. Of particular importance will be to confirm the hypothesis that the AEC of neonates who subsequently develop childhood asthma differ from AEC of children who do not develop asthma [1]. In addition it will be important to ascertain whether neonatal AEC function is associated with established risk factors for childhood asthma, e.g. parental asthma, maternal smoking and maternal diet. Indeed we are currently using the methodology developed and reported here in an ongoing birth cohort study that is aimed at addressing some of these issues.

Previous studies of bronchial AEC in children have been largely opportunistic and in highly selected groups for the most part involving the sampling of anaesthetised subjects in order to access the lower airway epithelium [20]-[22]. Clearly such methods are not viable in newborn infants and we believe that the method described here offers a simple acceptable alternative that could be applied to large scale epidemiological studies. Whilst the nose is an attractive source of AEC, there is active debate as to whether nasal AEC are valid surrogates for bronchial AEC [15], [23]. We previously reported identical epithelial morphology and immunostaining in paired cultures of nasal and bronchial AEC from 30 adults and 5 children [15]. Furthermore, release of several inflammatory mediators from nasal and bronchial AEC was positively correlated. More recently we have replicated these studies in a wholly paediatric population [16]. These findings suggest that the nasal and bronchial airway epithelial cells of newborn infants are likely to be similar. It is plausible that the constant postnatal exposure of nasal epithelial cells *in vivo* to a higher burden of environmental pollutants and pathogens relative to bronchial AEC leads to a differential up-regulation of inflammatory mediators with increasing age. Opportunities to access the lower airways of neonates for research purposes are exceptionally rare and consequently a study comparing directly nasal and bronchial AEC in the same individuals is highly unlikely to be feasible. We believe that our method of obtaining AEC from neonates is a pragmatic compromise and at the very least neonatal nasal AEC will provide a convenient model to investigate the pathogenesis of allergic rhinitis.

We have demonstrated both basal expression and dose dependent upregulation of the neutrophilic chemokine IL-8 in nasal AEC cultures with both a pro inflammatory (IL-1β/TNF-α) and allergenic (HDM) stimulus. This observation demonstrates that even in newborn infants, airway epithelial cells have the potential to function as components of the innate immune response and direct adaptive immune responses to pathogens and allergens. In this study IL-8 was used as an easily quantified exemplar cytokine. Further work is required to quantify the secretion of other cytokines, chemokines and growth factors by neonatal nasal AEC stimulated with a range of asthma-relevant cytokines such as IL-4 or IL-13.

In summary, we describe a safe, minimally invasive and reliable method of culturing AEC from neonates suitable for functional cell analysis and amenable to population based studies. This methodology offers a unique opportunity to study “naïve” AEC and may prove useful in elucidating the early origins of respiratory disease.
